# Correction: Semantic representation of neural circuit knowledge in *Caenorhabditis elegans*

**DOI:** 10.1186/s40708-024-00226-x

**Published:** 2024-05-15

**Authors:** Sharan J. Prakash, Kimberly M. Van Auken, David P. Hill, Paul W. Sternberg

**Affiliations:** 1 Division of Biology and Biological Engineering, California Institute of Technology, Pasadena, CA 91125 USA; 2 The Jackson Laboratory, Bar Harbor, ME 04609 USA

**Correction: Brain Informatics (2023) 10:30** 10.1186/s40708-023-00208-5

In this article [1], an error occurred during the production process regarding proper referencing, such that the in-text references do not correctly map to the reference list at the end of the article. In addition, three citations in the text did not appear in the reference list, and one reference item (the second item in the original list) appears incorrectly. Listed below are a corrected reference list, and a table listing all citations in order of appearance in the original publication, along with their corresponding corrections where necessary. A revised copy of the manuscript containing all corrections is available from corresponding author upon request.

**Corrected Reference List**

1. Alon U (2007) Network motifs: Theory and experimental approaches. Nat Rev Genet 8:450–461. doi: 10.1038/nrg2102.
2. Ashburner M, Ball CA, Blake JA, Botstein D, Butler H, Cherry JM, Davis AP, Dolinski K, Dwight SS, Eppig JT, Harris MA, Hill DP, Issel-Tarver L, Kasarskis A, Lewis S, Matese JC, Richardson JE, Ringwald M, Rubin GM, Sherlock G (2000) Gene Ontology: tool for the unification of biology. Nat Genet 25:25–29. doi: 10.1038/75556.
3. Banerjee N, Bhattacharya R, Gorczyca M, Collins KM, Francis MM (2017) Local neuropeptide signaling modulates serotonergic transmission to shape the temporal organization of *C. elegans* egg-laying behavior. PLoS Genet 13:e1006697. doi: 10.1371/journal.pgen.1006697.
4. Bany IA, Dong MQ, Koelle MR (2003) Genetic and cellular basis for acetylcholine inhibition of *Caenorhabditis elegans* egg-laying behavior. J Neurosci 23:8060–8069. doi: 10.1523/jneurosci.23-22-08060.2003.
5. Bargmann CI (1993) Genetic and Cellular Analysis of Behavior in *C. elegans*. Annu Rev Neurosci 16:47–71. doi: 10.1146/annurev.ne.16.030193.000403.
6. Bargmann CI (2012) Beyond the connectome: How neuromodulators shape neural circuits. BioEssays 34:458–465. doi: https://doi.org/10.1002/bies.201100185.
7. Bargmann CI, Avery L (1995) Laser killing of cells in *Caenorhabditis elegans*. Methods Cell Biol 48:225–250. doi: 10.1016/s0091-679x(08)61390-4.
8. Bargmann CI, Horvitz HR (1991) Control of larval development by chemosensory neurons in *Caenorhabditis elegans*. Science 251:1243–1246. doi: 10.1126/science.2006412.
9. Bastiani CA, Gharib S, Simon MI, Sternberg PW (2003) *Caenorhabditis elegans* Gαq Regulates Egg-Laying Behavior via a PLCβ-Independent and Serotonin-Dependent Signaling Pathway and Likely Functions Both in the Nervous System and in Muscle. Genetics 165:1805–1822. doi: 10.1093/genetics/165.4.1805.
10. Bellemer A, Hirata T, Romero MF, Koelle MR (2011) Two types of chloride transporters are required for GABA(A) receptor-mediated inhibition in *C. elegans*. EMBO J 30:1852–1863. doi: 10.1038/emboj.2011.83.
11. Bentley B, Branicky R, Barnes CL, Chew YL, Yemini E, Bullmore ET, Vértes PE, Schafer WR (2016) The Multilayer Connectome of *Caenorhabditis elegans*. PLoS Comput Biol 12:e1005283. doi: 10.1371/journal.pcbi.1005283.
12. Branicky R, Miyazaki H, Strange K, Schafer WR (2014) The voltage-gated anion channels encoded by clh-3 regulate egg laying in *C. elegans* by modulating motor neuron excitability. J Neurosci 34:764–775. doi: 10.1523/JNEUROSCI.3112-13.2014.
13. Bretscher AJ, Busch KE, De Bono M (2008) A carbon dioxide avoidance behavior is integrated with responses to ambient oxygen and food in *Caenorhabditis elegans*. Proc Nat Acad of Sci of the USA 105:8044–8049. doi: 10.1073/pnas.0707607105.
14. Bretscher AJ, Kodama-Namba E, Busch KE, Murphy RJ, Soltesz Z, Laurent P, de Bono M (2011) Temperature, oxygen, and salt-sensing neurons in *C. elegans* are carbon dioxide sensors that control avoidance behavior. Neuron 69:1099–1113. doi: 10.1016/j.neuron.2011.02.023.
15. Brundage L, Avery L, Katz A, Kim U-J, Mendel JE, Sternberg PW, Simon MI (1996) Mutations in a *C. elegans* Gqα Gene Disrupt Movement, Egg Laying, and Viability. Neuron 16:999–1009. doi: https://doi.org/10.1016/S0896-6273(00)80123-3.
16. Carnell L, Illi J, Hong SW, McIntire SL (2005) The G-protein-coupled serotonin receptor SER-1 regulates egg laying and male mating behaviors in *Caenorhabditis elegans*. J Neurosci 25:10671–10681. doi: 10.1523/JNEUROSCI.3399-05.2005.
17. Carrillo MA, Guillermin ML, Rengarajan S, Okubo RP, Hallem EA (2013) O_2_-sensing neurons control CO_2_ response in *C. elegans*. J Neurosci 33:9675–9683. doi: 10.1523/JNEUROSCI.4541-12.2013.
18. Chalfie M, Sulston JE, White JG, Southgate E, Nichol Thomson J, Brenner S (1985) The neural circuit for touch sensitivity in *Caenorhabditis elegans*. J Neurosci 5:956–964. doi: 10.1523/jneurosci.05-04-00956.1985.
19. Chan LE, Thessen AE, Duncan WD, Matentzoglu N, Schmitt C, Grondin C, Vasilevsky N, McMurry JA, Robinson PN, Mungall CJ (2022) The Environmental Conditions, Treatments, and Exposures Ontology (ECTO): connecting toxicology and exposure to human health and beyond. J Biomed Semantics 14:3. doi: 10.1186/s13326-023-00283-x
20. Cheung BHH, Arellano-Carbajal F, Rybicki I, de Bono M (2004) Soluble guanylate cyclases act in neurons exposed to the body fluid to promote *C. elegans* aggregation behavior. Curr Biol 14:1105–1111. doi: 10.1016/j.cub.2004.06.027.
21. Choi J-H, Horowitz LB, Ringstad N (2021) Opponent vesicular transporters regulate the strength of glutamatergic neurotransmission in a *C. elegans* sensory circuit. Nat Commun 12:6334. doi: 10.1038/s41467-021-26575-3.
22. Chung SH, Sun L, Gabel CV (2013) In vivo neuronal calcium imaging in *C. elegans*. J Vis Exp : JoVE. doi: 10.3791/50357.
23. Clark DA, Gabel CV, Gabel H, Samuel ADT (2007) Temporal activity patterns in thermosensory neurons of freely moving *Caenorhabditis elegans* encode spatial thermal gradients. J Neurosci 27:6083–6090. doi: 10.1523/JNEUROSCI.1032-07.2007.
24. Coates JC, de Bono M (2002) Antagonistic pathways in neurons exposed to body fluid regulate social feeding in *Caenorhabditis elegans*. Nature 419:925–929. doi: 10.1038/nature01170.
25. Collins KM, Bode A, Fernandez RW, Tanis JE, Brewer JC, Creamer MS, Koelle MR (2016) Activity of the *C. elegans* egg-laying behavior circuit is controlled by competing activation and feedback inhibition. eLife 5:1–24. doi: 10.7554/eLife.21126.
26. Collins KM, Koelle MR (2013) Postsynaptic ERG potassium channels limit muscle excitability to allow distinct egg-laying behavior states in *Caenorhabditis elegans*. J Neurosci 33:761–775. doi: 10.1523/JNEUROSCI.3896-12.2013.
27. Court R, Costa M, Pilgrim C, Millburn G, Holmes A, McLachlan A, Larkin A, Matentzoglu N, Kir H, Parkinson H, Brown NH, O’Kane CJ, Armstrong JD, Jefferis GSXE, Osumi-Sutherland D (2023) Virtual Fly Brain—An interactive atlas of the *Drosophila* nervous system. Front in Physiol 14.
28. Emtage L, Aziz-Zaman S, Padovan-Merhar O, Horvitz HR, Fang-Yen C, Ringstad N (2012) IRK-1 potassium channels mediate peptidergic inhibition of *Caenorhabditis elegans* serotonin neurons via a Go signaling pathway. J Neurosci 32:16285–16295. doi: 10.1523/JNEUROSCI.2667-12.2012.
29. Fenk LA, de Bono M (2015) Environmental CO_2_ inhibits *Caenorhabditis elegans* egg-laying by modulating olfactory neurons and evokes widespread changes in neural activity. Proc Nat Acad of Sci USA 112:E3525-34. doi: 10.1073/pnas.1423808112.
30. Ghosh DD, Nitabach MN, Zhang Y, Harris G (2017) Multisensory integration in *C. elegans*. Curr Opin in Neurobiol 43:110–118. doi: https://doi.org/10.1016/j.conb.2017.01.005.
31. Giglio M, Tauber R, Nadendla S, Munro J, Olley D, Ball S, Mitraka E, Schriml LM, Gaudet P, Hobbs ET, Erill I, Siegele DA, Hu JC, Mungall C, Chibucos MC (2019) ECO, the Evidence & Conclusion Ontology: community standard for evidence information. Nucleic Acids Res 47:D1186–D1194. doi: 10.1093/nar/gky1036.
32. Good BM, Van Auken K, Hill DP, Mi H, Carbon S, Balhoff JP, Albou L-P, Thomas PD, Mungall CJ, Blake JA, D’Eustachio P (2021) Reactome and the Gene Ontology: digital convergence of data resources. Bioinformatics 37:3343–3348. doi: 10.1093/bioinformatics/btab325.
33. Gray JM, Karow DS, Lu H, Chang AJ, Chang JS, Ellis RE, Marletta MA, Bargmann CI (2004) Oxygen sensation and social feeding mediated by a *C. elegans* guanylate cyclase homologue. Nature 430:317–322. doi: 10.1038/nature02714.
34. Guo ZV, Hart AC, Ramanathan S (2009) Optical interrogation of neural circuits in *Caenorhabditis elegans*. Nat Methods 6:891–896. doi: 10.1038/nmeth.1397.
35. Hallem EA, Sternberg PW (2008) Acute carbon dioxide avoidance in *Caenorhabditis elegans*. Proc Natl Acad Sci USA 105:8038–8043. doi: 10.1073/pnas.0707469105.
36. Harbinder S, Tavernarakis N, Herndon LA, Kinnell M, Xu SQ, Fire A, Driscoll M (1997) Genetically targeted cell disruption in *Caenorhabditis elegans*. Proc Natl Acad Sci USA 94:13128–13133. doi: 10.1073/pnas.94.24.13128.
37. Hastings J, Owen G, Dekker A, Ennis M, Kale N, Muthukrishnan V, Turner S, Swainston N, Mendes P, Steinbeck C (2016) ChEBI in 2016: Improved services and an expanding collection of metabolites. Nucleic Acids Res 44:D1214-9. doi: 10.1093/nar/gkv1031.
38. Husson SJ, Gottschalk A, Leifer AM (2013) Optogenetic manipulation of neural activity in *C. elegans*: from synapse to circuits and behaviour. Biol Cell 105:235–250. doi: 10.1111/boc.201200069.
39. Jarrell TA, Wang Y, Bloniarz AE, Brittin CA, Xu M, Thomson JN, Albertson DG, Hall DH, Emmons SW (2012) The connectome of a decision-making neural network. Science (New York, NY) 337:437–444. doi: 10.1126/science.1221762.
40. Juanes Cortés B, Vera-Ramos JA, Lovering RC, Gaudet P, Laegreid A, Logie C, Schulz S, Roldán-García MDM, Kuiper M, Fernández-Breis JT (2021) Formalization of gene regulation knowledge using ontologies and gene ontology causal activity models. Biochim Biophys Acta Gene Regul Mech 1864:194766. doi: 10.1016/j.bbagrm.2021.194766.
41. Kim J, Poole DS, Waggoner LE, Kempf A, Ramirez DS, Treschow PA, Schafer WR (2001) Genes affecting the activity of nicotinic receptors involved in *Caenorhabditis elegans* egg-laying behavior. Genetics 157:1599–1610. doi: 10.1093/genetics/157.4.1599.
42. Kimura KD, Miyawaki A, Matsumoto K, Mori I (2004) The *C. elegans* thermosensory neuron AFD responds to warming. Curr Biol 14:1291–1295. doi: 10.1016/j.cub.2004.06.060.
43. Koelle MR, Horvitz HR (1996) EGL-10 regulates G protein signaling in the *C. elegans* nervous system and shares a conserved domain with many mammalian proteins. Cell 84:115–125. doi: 10.1016/s0092-8674(00)80998-8.
44. Koopmans F, van Nierop P, Andres-Alonso M, Byrnes A, Cijsouw T, Coba MP, Cornelisse LN, Farrell RJ, Goldschmidt HL, Howrigan DP, Hussain NK, Imig C, de Jong APH, Jung H, Kohansalnodehi M, Kramarz B, Lipstein N, Lovering RC, MacGillavry H, Mariano V, Mi H, Ninov M, Osumi-Sutherland D, Pielot R, Smalla K-H, Tang H, Tashman K, Toonen RFG, Verpelli C, Reig-Viader R, Watanabe K, van Weering J, Achsel T, Ashrafi G, Asi N, Brown TC, De Camilli P, Feuermann M, Foulger RE, Gaudet P, Joglekar A, Kanellopoulos A, Malenka R, Nicoll RA, Pulido C, de Juan-Sanz J, Sheng M, Südhof TC, Tilgner HU, Bagni C, Bayés À, Biederer T, Brose N, Chua JJE, Dieterich DC, Gundelfinger ED, Hoogenraad C, Huganir RL, Jahn R, Kaeser PS, Kim E, Kreutz MR, McPherson PS, Neale BM, O’Connor V, Posthuma D, Ryan TA, Sala C, Feng G, Hyman SE, Thomas PD, Smit AB, Verhage M (2019) SynGO: An Evidence-Based, Expert-Curated Knowledge Base for the Synapse. Neuron 103:217-234.e4. doi: https://doi.org/10.1016/j.neuron.2019.05.002.
45. Kopchock RJ, Ravi B, Bode A, Collins KM (2021) The Sex-Specific VC Neurons Are Mechanically Activated Motor Neurons That Facilitate Serotonin-Induced Egg Laying in *C. elegans.* J Neurosci 41:3635–3650. doi: 10.1523/JNEUROSCI.2150-20.2021.
46. Larsch J, Flavell SW, Liu Q, Gordus A, Albrecht DR, Bargmann CI (2015) A Circuit for Gradient Climbing in *C. elegans* Chemotaxis. Cell Rep 12:1748–1760. doi: 10.1016/j.celrep.2015.08.032.
47. Le Novère N, Hucka M, Mi H, Moodie S, Schreiber F, Sorokin A, Demir E, Wegner K, Aladjem MI, Wimalaratne SM, Bergman FT, Gauges R, Ghazal P, Kawaji H, Li L, Matsuoka Y, Villéger A, Boyd SE, Calzone L, Courtot M, Dogrusoz U, Freeman TC, Funahashi A, Ghosh S, Jouraku A, Kim S, Kolpakov F, Luna A, Sahle S, Schmidt E, Watterson S, Wu G, Goryanin I, Kell DB, Sander C, Sauro H, Snoep JL, Kohn K, Kitano H (2009) The Systems Biology Graphical Notation. Nat Biotechnol 27:735–741. doi: 10.1038/nbt.1558.
48. Lee RYN, Sternberg PW (2003) Building a cell and anatomy ontology of *Caenorhabditis elegans*. Comp Funct Genomics 4:121–126. doi: 10.1002/cfg.248.
49. Leifer AM, Fang-Yen C, Gershow M, Alkema MJ, Samuel ADT (2011) Optogenetic manipulation of neural activity in freely moving *Caenorhabditis elegans*. Nat Methods 8:147–152. doi: 10.1038/nmeth.1554.
50. Lickteig KM, Duerr JS, Frisby DL, Hall DH, Rand JB, Miller DM 3rd (2001) Regulation of neurotransmitter vesicles by the homeodomain protein UNC-4 and its transcriptional corepressor UNC-37/groucho in *Caenorhabditis elegans* cholinergic motor neurons. J Neurosci 21:2001–2014. doi: 10.1523/JNEUROSCI.21-06-02001.2001.
51. Liu KS, Sternberg PW (1995) Sensory regulation of male mating behavior in *Caenorhabditis elegans*. Neuron 14:79–89. doi: https://doi.org/10.1016/0896-6273(95)90242-2.
52. MacOsko EZ, Pokala N, Feinberg EH, Chalasani SH, Butcher RA, Clardy J, Bargmann CI (2009) A hub-and-spoke circuit drives pheromone attraction and social behaviour in *C. elegans*. Nature 458:1171–1175. doi: 10.1038/nature07886.
53. Marder E (2012) Neuromodulation of neuronal circuits: back to the future. Neuron 76:1–11. doi: 10.1016/j.neuron.2012.09.010.
54. Milyaev N, Osumi-Sutherland D, Reeve S, Burton N, Baldock RA, Armstrong JD (2012) The Virtual Fly Brain browser and query interface. Bioinformatics 28:411–415. doi: 10.1093/bioinformatics/btr677.
55. Mori I, Ohshima Y (1995) Neural regulation of thermotaxis in *Caenorhabditis elegans*. Nature 376:344–348. doi: 10.1038/376344a0.
56. Okkema PG, Harrison SW, Plunger V, Aryana A, Fire A (1993) Sequence requirements for myosin gene expression and regulation in *Caenorhabditis elegans*. Genetics 135:385–404. doi: 10.1093/genetics/135.2.385.
57. Pokala N, Liu Q, Gordus A, Bargmann CI (2014) Inducible and titratable silencing of *Caenorhabditis elegans* neurons in vivo with histamine-gated chloride channels. Proc Nat Acad of Sci USA 111:2770–2775. doi: 10.1073/pnas.1400615111.
58. Ravi B, Garcia J, Collins KM (2018) Homeostatic feedback modulates the development of two-state patterned activity in a model serotonin motor circuit in *Caenorhabditis elegans.* J Neurosci 38:6283–6298. doi: 10.1523/JNEUROSCI.3658-17.2018.
59. Reigl M, Alon U, Chklovskii DB (2004) Search for computational modules in the *C. elegans* brain. BMC Biol 2:25. doi: 10.1186/1741-7007-2-25.
60. Richmond JE, Davis WS, Jorgensen EM (1999) UNC-13 is required for synaptic vesicle fusion in *C. elegans*. Nat Neurosci 2:959–964. doi: 10.1038/14755.
61. Schindelman G, Fernandes JS, Bastiani CA, Yook K, Sternberg PW (2011) Worm Phenotype Ontology: Integrating phenotype data within and beyond the *C. elegans* community. BMC Bioinformatics 12:32. doi: 10.1186/1471-2105-12-32.
62. Shannon P, Markiel A, Ozier O, Baliga NS, Wang JT, Ramage D, Amin N, Schwikowski B, Ideker T (2003) Cytoscape: a software environment for integrated models of biomolecular interaction networks. Genome Res 13:2498–2504. doi: 10.1101/gr.1239303.
63. Shen Y, Wen Q, Liu H, Zhong C, Qin Y, Harris G, Kawano T, Wu M, Xu T, Samuel AD, Zhang Y (2016) An extrasynaptic GABAergic signal modulates a pattern of forward movement in *Caenorhabditis elegans*. eLife 5. doi: 10.7554/eLife.14197.
64. Shine JM, Müller EJ, Munn B, Cabral J, Moran RJ, Breakspear M (2021) Computational models link cellular mechanisms of neuromodulation to large-scale neural dynamics. Nat Neurosci 24:765–776. doi: 10.1038/s41593-021-00824-6.
65. Shyn SI, Kerr R, Schafer WR (2003) Serotonin and Go Modulate Functional States of Neurons and Muscles Controlling *C. elegans* Egg-Laying Behavior. Curr Biol 13:1910–1915. doi: 10.1016/j.cub.2003.10.025.
66. Smith B, Ceusters W, Klagges B, Köhler J, Kumar A, Lomax J, Mungall C, Neuhaus F, Rector AL, Rosse C (2005) Relations in biomedical ontologies. Genome Biol 6:R46. doi: 10.1186/gb-2005-6-5-r46.
67. Speese S, Petrie M, Schuske K, Ailion M, Ann K, Iwasaki K, Jorgensen EM, Martin TFJ (2007) UNC-31 (CAPS) is required for dense-core vesicle but not synaptic vesicle exocytosis in *Caenorhabditis elegans*. J Neurosci  27:6150–6162. doi: 10.1523/JNEUROSCI.1466-07.2007.
68. Srinivasan J, von Reuss SH, Bose N, Zaslaver A, Mahanti P, Ho MC, O’Doherty OG, Edison AS, Sternberg PW, Schroeder FC (2012) A modular library of small molecule signals regulates social behaviors in *Caenorhabditis elegans*. PLoS Biol 10:1–14. doi: 10.1371/journal.pbio.1001237.
69. Sweeney ST, Broadie K, Keane J, Niemann H, O’Kane CJ (1995) Targeted expression of tetanus toxin light chain in *Drosophila* specifically eliminates synaptic transmission and causes behavioral defects. Neuron 14:341–351. doi: https://doi.org/10.1016/0896-6273(95)90290-2.
70. Tanis JE, Bellemer A, Moresco JJ, Forbush B, Koelle MR (2009) The potassium chloride cotransporter KCC-2 coordinates development of inhibitory neurotransmission and synapse structure in *Caenorhabditis elegans*. J Neurosci 29:9943–9954. doi: 10.1523/JNEUROSCI.1989-09.2009.
71. The Gene Ontology Consortium (2021) The Gene Ontology resource: enriching a GOld mine. Nucleic Acids Res 49:D325–D334. doi: 10.1093/nar/gkaa1113.
72. Thomas PD, Hill DP, Mi H, Osumi-Sutherland D, Van Auken K, Carbon S, Balhoff JP, Albou L-P, Good B, Gaudet P, Lewis SE, Mungall CJ (2019) Gene Ontology Causal Activity Modeling (GO-CAM) moves beyond GO annotations to structured descriptions of biological functions and systems. Nat Genet 51:1429–1433. doi: 10.1038/s41588-019-0500-1.
73. Trent C, Tsuing N, Horvitz HR (1983) Egg-laying defective mutants of the nematode *Caenorhabditis elegans*. Genetics 104:619–647. doi: 10.1093/genetics/104.4.619.
74. Waggoner LE, Zhou GT, Schafer RW, Schafer WR (1998) Control of alternative behavioral states by serotonin in *Caenorhabditis elegans*. Neuron 21:203–214. doi: 10.1016/S0896-6273(00)80527-9.
75. Wen X, Chen Y-H, Li R, Ge M-H, Yin S-W, Wu J-J, Huang J-H, Liu H, Wang P-Z, Gross E, Wu Z-X (2020) Signal Decoding for Glutamate Modulating Egg Laying Oppositely in *Caenorhabditis elegans* under Varied Environmental Conditions. iScience 23:101588. doi: https://doi.org/10.1016/j.isci.2020.101588.
76. Zhang M, Chung SH, Fang-Yen C, Craig C, Kerr RA, Suzuki H, Samuel ADT, Mazur E, Schafer WR (2008) A Self-Regulating Feed-Forward Circuit Controlling *C. elegans* Egg-Laying Behavior. Curr Biol 18:1445–1455. doi: 10.1016/j.cub.2008.08.047.

**Table of Corrections**

**Table:** 

Source	Current	Correction
Main text	[8,9,18,49]	[7, 8, 18, 51]
Main text	[34,65]	[36, 38]
Main text	[7]	[5]
Main text	[72]	[74]
Main text	[36,55]	[38, 57]
Main text	[22]	[22]
Main text	[60]	[63]
Main text	[6,11,51]	[6, 11, 53]
Main text	[70]	[72]
Main text	[44]	[47]
Main text	[63]	[66]
Main text	[3,30,69]	[2,31,71]
Main text	[30]	[31]
Main text	[3,69]	[2, 71]
Main text	[19]	[19]
Main text	[45]	[48]
Main text	[63]	[66]
Main text	[19]	[19]
Main text	[18]	[18]
Main text	[33]	[34]
Main text	[13]	[14]
Main text	[42]	[45]
Main text	[58]	[60]
Main text	[64]	[67]
Main text	[21]	[21]
Main text	[70]	[72]
Main text	[28]	[29]
Main text	[74]	[75]
Main text	[29]	[30]
Main text	[13]	[14]
Main text	[63]	[66]
Main text	[42]	[45]
Main text	[61]	[64]
Main text	[55]	[57]
Main text	[67]	[69]
Main text	[25]	[25]
Main text	[13]	[14]
Main text	Schindelman et al. (2011)	[61]
Main text	[38, 70]	[40, 72]
Main text	[41]	[44]
Main text	[31]	[32]
Main text	[43]	[46]
Main text	[29]	[30]
Main text	[13]	[14]
Main text	[62]	[65]
Main text	[45]	[48]
Main text	[26, 52]	[27, 54]
Main text	[59]	[62]
Main text	[28]	[29]
Main text	[73]	[75]
Main text	[1, 37, 57]	[1, 39, 59]
Table 4A	Waggoner et al. [72]	Waggoner et al. [74]
Table 4B	Bany et al. [5]	Bany et al. [4]
Table 4B	[48]	[50]
Table 4C	Banerjee et al. [4]	Banerjee et al. [3]
Table 4D	Carnell et al. [15]	Carnell et al.[16]
Table 4D	[54]	[56]
Table 4D	[14,71,72, Bastiani et al. 2003, 62]	[9, 15, 65, 73, 74]
Table 4E	Carillo et al. [16]	Carrillo et al. [17]
Table 4E	[50]	[52]
Table 4E	[20, 32]	[20,33]
Table 4F	Bretscher et al. [13]	Bretscher et al. [14]
Table 4F	[58]	[60]
Table 4F	[23, 39, 53]	[25, 42, 55]
Table 4G	Collins et al. [25]	Collins et al. [25]
Table 4G	[27]	[28]
Table 4H	Kopchock et al. [42]	Kopchock et al. [45]
Table 4H	[25, 56]	[25, 58]
Table 4H	[5, 74]	[4, 75]
Table 4J	Choi et al. [21]	Choi et al. [21]
Table 4K	Choi et al. [21]	Choi et al. [21]
Table 5A	Bretscher et al. [13]	Bretscher et al. [14]
Table 5A	[12]	[13]
Table 5A	[24]	[24]
Table 5A	[35]	[35]
Table 5B	Kopchock et al. [42]	Kopchock et al. [45]
Table 5B	[3, 25]	[25, 26]
Table 5B	[Collins and Koelle (2013), 25]	[25, 26]
Table 5C	Branicky et al. [11]	Branicky et al. [12]
Table 5C	[27, 46]	[28, 49]
Table 5C	[46]	[49]
Table 5D	Emtage et al. [27]	Emtage et al. [28]
Table 5D	[40]	[43]
Table 5E	Collins et al. [25]	Collins et al. [25]
Table 5E	[10, 68]	[10, 70]
Table 5F	Bretscher et al. [13]	Bretscher et al. [14]
Table 5G	Shyn et al. [62]	Shyn et al. [65]
Figure 2A	Waggoner et al. [72]	Waggoner et al. [74]
Figure 2B	[5]	[4]
Figure 2C	[4]	[3]
Figure 3A	Carnell et al. [15]	Carnell et al. [16]
Figure 3B	[16]	Carrillo et al. [17]
Figure 3C	[13]	Bretscher et al. [14]
Figure 4A	[25]	[25]
Figure 4B	[42]	[45]
Figure 4C	Choi et al. [21]	Choi et al. [21]
Figure 5	[1, 15, 25, 28, 42]	[16, 25, 29, 41, 45]
