# Supplementary figures and images for: Semantic representation of neural circuit knowledge in Caenorhabditis elegans
Source: Brain Inform. 2023 Nov 10;10(1):30. doi: 10.1186/s40708-023-00208-5 (PMC10638142; doi:10.1186/s40708-023-00208-5)

## Slide 1
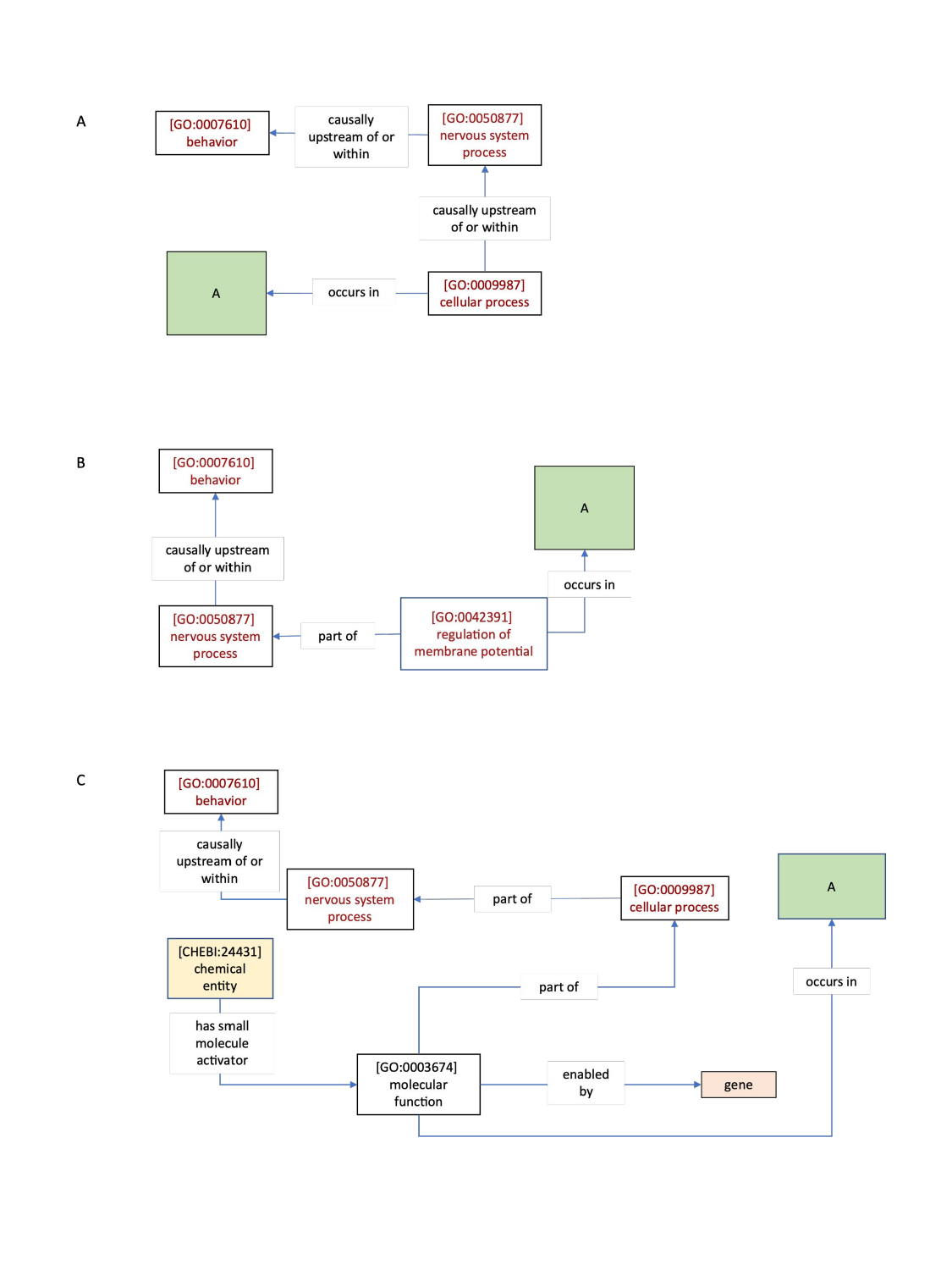

## Slide 2
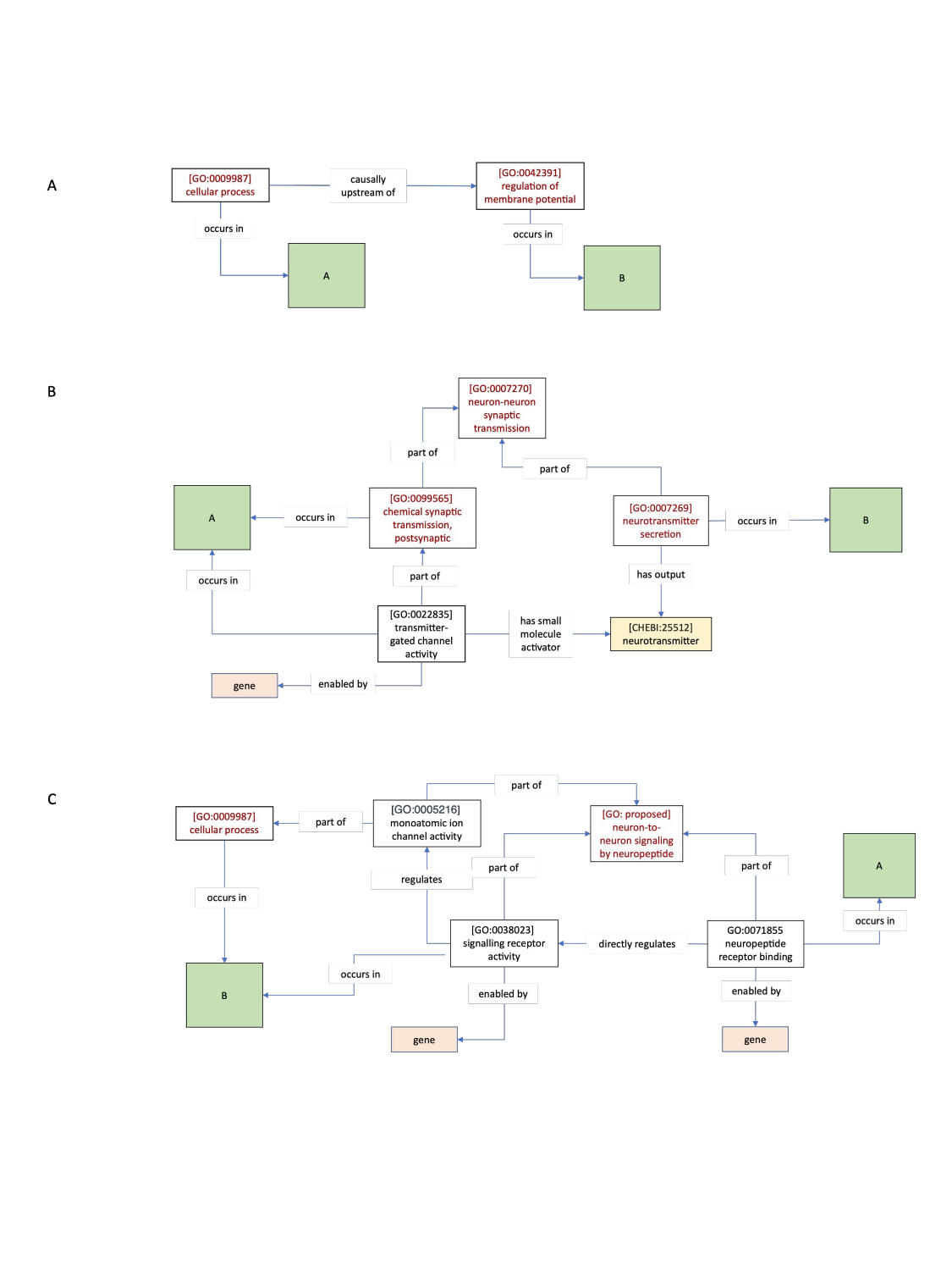

## Slide 3
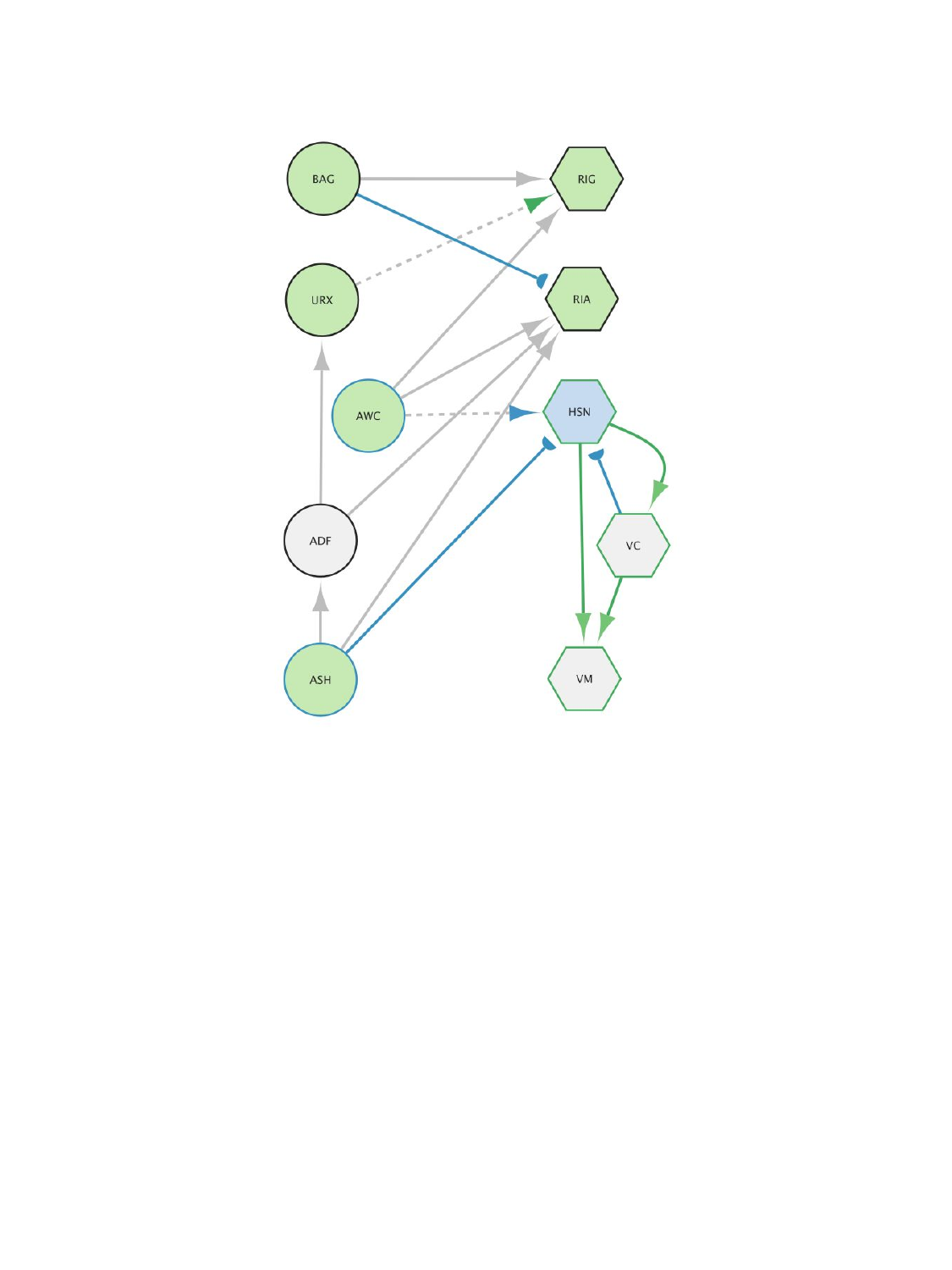

Supplement: Supplementary file 1 — Additional file 1: Figure S1. Curation templates for experimental results linking neurons to behavior. Each GO term and each relation represents the most generic (highest level) term that is suitable for the model. Authors can populate a model with either these relations, or any of its child terms. High level GO biological process terms can also be expanded to include an arbitrary number of constituent GO molecular functions or GO biological processes. The following labels describe the causal flow described by each model, and the corresponding type of experiment. A Neuron to Behavior (ablation) B Neuron to Behavior (activation/inhibition). C Input to Neuron to Behavior (rescue). Figure S2. Curation templates for experimental results inputs describing functional connections between neurons, whether A mechanism agnostic (neuron to neuron), B via synapses, or C) extra-synaptic neuropeptide signalling. Terms without IDs are novel terms. Figure S3. Cytoscape rendering of the same neural circuit as in Fig. 8 (this manuscript). Filled cells are responsive to CO2 (green cells are activated, blue cells are inhibited). Outlined cells are involved in egg-laying (green outlined cells promote, and blue outlined cells inhibit egg-laying, respectively. [file 40708_2023_208_MOESM1_ESM.pptx]
